# Persistence of Smoking-Induced Dysregulation of MiRNA Expression in the Small Airway Epithelium Despite Smoking Cessation

**DOI:** 10.1371/journal.pone.0120824

**Published:** 2015-04-17

**Authors:** Guoqing Wang, Rui Wang, Yael Strulovici-Barel, Jacqueline Salit, Michelle R. Staudt, Joumana Ahmed, Ann E. Tilley, Jenny Yee-Levin, Charleen Hollmann, Ben-Gary Harvey, Robert J. Kaner, Jason G. Mezey, Sriram Sridhar, Sreekumar G. Pillai, Holly Hilton, Gerhard Wolff, Hans Bitter, Sudha Visvanathan, Jay S. Fine, Christopher S. Stevenson, Ronald G. Crystal

**Affiliations:** 1 Department of Genetic Medicine, Weill Cornell Medical College, New York, New York, United States of America; 2 Division of Pulmonary and Critical Care Medicine, Department of Medicine, Weill Cornell Medical College, New York, New York, United States of America; 3 Department of Biological Statistics and Computational Biology, Cornell University, Ithaca, New York, United States of America; 4 Hoffman-La Roche, Inc, Nutley, New Jersey, United States of America; Institute of Lung Biology and Disease (iLBD), Helmholtz Zentrum München, GERMANY

## Abstract

Even after quitting smoking, the risk of the development of chronic obstructive pulmonary disease (COPD) and lung cancer remains significantly higher compared to healthy nonsmokers. Based on the knowledge that COPD and most lung cancers start in the small airway epithelium (SAE), we hypothesized that smoking modulates miRNA expression in the SAE linked to the pathogenesis of smoking-induced airway disease, and that some of these changes persist after smoking cessation. SAE was collected from 10^th^ to 12^th^ order bronchi using fiberoptic bronchoscopy. Affymetrix miRNA 2.0 arrays were used to assess miRNA expression in the SAE from 9 healthy nonsmokers and 10 healthy smokers, before and after they quit smoking for 3 months. Smoking status was determined by urine nicotine and cotinine measurement. There were significant differences in the expression of 34 miRNAs between healthy smokers and healthy nonsmokers (p<0.01, fold-change >1.5), with functions associated with lung development, airway epithelium differentiation, inflammation and cancer. After quitting smoking for 3 months, 12 out of the 34 miRNAs did not return to normal levels, with Wnt/β-catenin signaling pathway being the top identified enriched pathway of the target genes of the persistent dysregulated miRNAs. In the context that many of these persistent smoking-dependent miRNAs are associated with differentiation, inflammatory diseases or lung cancer, it is likely that persistent smoking-related changes in SAE miRNAs play a role in the subsequent development of these disorders.

## Introduction

Cigarette smoking, with its >4000 compounds and 10^14^ oxidants per puff, is the major cause of two major lung disorders, chronic obstructive pulmonary disease (COPD) and bronchogenic carcinoma [1–3]. COPD and most cases of lung cancer start in the small airway epithelium (SAE), the cell population lining the airways ≥6^th^ generations [4–6]. While cessation of smoking reduces the risk for smoking-induced lung disease, epidemiologic studies have shown that the risk never disappears [7,8], suggesting that there are lung-related biologic processes that continue despite smoking cessation. There is extensive data from our laboratory and others demonstrating that smoking is associated with up- and down-regulation of many genes in the airway epithelium [9–12]. Interestingly, and consistent with the epidemiologic data, ex-smokers continue to have persistent up- and down-regulation of many genes, despite smoking cessation [11,13].

There are likely a number of explanations for this, as cigarette smoke is complex, and there are multiple pathways by which smoking may influence airway epithelial gene expression [3,14]. Importantly, smoking affects the expression of many genes in the airway epithelium, suggesting that the biologic processes involved, likely have a broad influence in gene expression. One such class of biologic processes are microRNAs (miRNA), small non-coding endogenous, single-stranded, 17 to 25 nucleotide-long RNAs that are generated by sequential processing from longer transcripts that contain a stem-loop [15]. These small regulatory RNAs modulate gene expression by binding to the 3’ end of target mRNAs, resulting in gene silencing through induction of mRNA cleavage or translational suppression [15,16]. Due to redundancy in self-complementary miRNA-mRNA binding, one miRNA often controls several potential mRNA targets, or alternately, one mRNA can be controlled by several miRNAs. In the lung, miRNA profiling studies have shown that miRNAs play a role in lung organogenesis [17–19] and that smoking can lead to the dysregulation of miRNAs in the large airway epithelium [20].

In the context of these considerations, we hypothesized that, not only does smoking modify the expression of miRNAs in the airway epithelium, but that with smoking cessation, a subset of these miRNAs continue to be dysregulated, with the consequences of persistent modification of the expression of several SAE genes despite smoking cessation. To assess this hypothesis, genome-wide levels of miRNA were assessed in the SAE from healthy nonsmokers, and healthy smokers before and after 3 months of smoking cessation. The data demonstrates that, not only does smoking alter the expression in the SAE of many miRNAs, but also many of these smoking-induced alterations in miRNA expression remain abnormally expressed after smoking cessation, with many associated with chronic airway inflammation or malignancy.

## Methods

### Ethics Statement

All individuals were evaluated and samples collected in the Weill Cornell NIH Clinical and Translational Science Center and Department of Genetic Medicine Clinical Research Facility under clinical protocols approved for this study by the Weill Cornell Medical College and New York/Presbyterian Hospital Institutional Review Boards (IRB) according to local and national IRB guidelines. All subjects gave their informed written consent prior to any clinical evaluations or procedures.

### Study Population

Nineteen study subjects, including 9 healthy nonsmokers and 10 healthy smokers, were assessed using protocols approved by the Weill Cornell Medical College IRB. In order to study the smoking cessation effect on small airway epithelial miRNA expression, the 10 healthy smokers were asked to quit smoking with the aid of varenicline (Chantix, targeting alpha-4 beta-2 nicotinic receptor in the brain) and counseling (see S1 Methods for inclusion/exclusion criteria). The SAE (10–12^th^ order) was collected by fiberoptic bronchoscopy by brushing as described previously (9). For all subjects, a baseline bronchoscopy was carried out in order to compare healthy nonsmokers to healthy smokers. For the healthy smokers, a second bronchoscopy was carried out 3 months after quitting smoking.

### miRNA Microarray Processing and Analysis

Following RNA extraction and sample quality assessment, miRNA microarray analyses were performed using Affymetrix miRNA 2.0 arrays (Affymetrix, Santa Clara CA). The raw data are available at the Gene Expression Omnibus (GEO) site (http://www.ncbi.nlm.nih.gov/geo, accession number for this dataset GSE 53519). The image files from Affymetrix miRNA 2.0 microarrays were processed in Partek Genomics Suite software version 6.6, 2012 (Partek Inc, St. Louis, MO). Two way ANOVA (gender was identified as a source of variation) followed by Fisher’s Least Significant Difference test was used to identify the differentially expressed miRNAs among groups. Additional details are provided in S1 Methods.

To quantitate the cessation response for each of the smoking-dependent miRNAs, the mean absolute expression levels of all healthy nonsmokers, healthy smokers before quitting, and healthy smokers after quitting smoking for 3 months were assessed as: % change = [(healthy smokers before smoking cessation—healthy smokers after 3-month smoking cessation)x100 / (healthy smokers before smoking cessation—healthy nonsmokers)].

To evaluate the global change of expression of these smoking-dependent miRNAs, a “smoking-dependent miRNA index” was created. For each miRNA, the expression quartile was calculated from the corresponding values in nonsmoker. The index was calculated for each subject as:

$$Smoking-dependent\;miRNA\;index=\;\overset{34}{\underset{n=1}{\sum }}E_{n}$$

where E1 has a value of 1 if the expression level for smoking up-regulated miRNA 1 was >3^rd^ quartile of all healthy nonsmokers, or <1^st^ quartile if this miRNA was a smoking down-regulated miRNA; E2 was the index for miRNA 2, etc.; the total, “n = 34”, is the number of all smoking-dependent miRNAs. The statistical significance of differences of index between the groups was determined using the Kruskal-Wallis test followed by Dunn’s test.

Direct prediction of the functions and associated diseases of the list of significantly changed miRNAs was carried out using Tool for Annotations of microRNAs (TAM; http://202.38.126.151/hmdd/tools/tam.html), which catalogs miRNAs into various categories. miRNA target gene prediction was carried out in Partek 6.6, which uses the TargetScan 6.2 database to search target genes. As not all genes are expressed in the SAE, the predicted-target gene list was filtered by “SAE genes” previously identified by deep sequencing (RNA-Seq) with expression level >0.125 RPKM (reads per kilobase of exon per million mapped reads) [21]. Pathway analysis of the target genes was performed in Ingenuity Pathway Analysis (IPA, Redwood City, CA).

The effect of smoking on the expression the miRNA target genes in the small airway epithelium were analyzed using our recently published Affymetrix Human Genome U133 plus 2.0 array dataset (16 healthy nonsmokers and 20 healthy smokers). The captured image data from the arrays were processed using the MAS5 algorithm [22]. For the genes with multiple probesets, only the one with highest average expression level was kept. For all 41 Wnt/β-catenin signaling pathway genes listed in S3 Table, 40 genes have matched probesets (none for Wnt3A). Principal component analysis was performed in Partek 6.6 using the default setting.

### Quantitative Real-Time PCR Validation

Quantitative real-time PCR (qRT-PCR, TaqMan; Life Technologies, Grand Island, NY) was used to confirm the differential expression of miRNAs (S1 Table). Small RNA RUN6B was used as endogenous control. Data were analyzed using the comparative C_T_ method. P values were determined using Mann-Whitney test.

### Assessment of miR-1246 Inhibition on Target Genes

Human primary airway epithelial cells (Lonza, Allendale, NJ) were cultured in 24 well plates (6×10^4^/well). After 1 day, the cells were transfected with miR-1246 inhibitor and negative microRNA inhibitor control (mirVana miRNA Inhibitor, Life Technologies, Grand Island, NY) at 66 nM concentration using Lipofectamine RNAiMAX reagent (Life Technologies, Grand Island, NY). Samples were collected after 36 hr for RNA extraction. TaqMan PCR was used to assess expression changes of 6 putative miR-1246 target genes, including DRK1A, GRHL1, GFHL2, GSK3B, CREB3L2 and PCTH1 (predicted by TargetScan website). 18S was used as endogenous control. Data were analyzed using the comparative C_T_ method.

## Results

### Expression of miRNAs in Healthy Smokers Compared with Healthy Nonsmokers

All the subjects were healthy based on self-reported history, physical exam, complete blood count, coagulation studies, liver function tests, urine studies, chest X-ray, EKG and pulmonary function tests (Table 1). All of the 10 smoking quitters were long term smokers and confirmed to be successful quitters by assessment of urine cotinine and nicotine (see S1 Methods for inclusion/exclusion criteria).

**Table 1:** Demographics of healthy nonsmokers, healthy smokers and healthy smokers after 3-month smoking cessation^1^.

Parameters	Healthy nonsmokers	Healthy smokers	
		Month 0	Month 3 (quitters)^2^
n	9	10	
Gender (male/female)	4/5	5/5	
Age (year)	44 ± 10	47 ± 7	
Race (B/W/O)^3^	6/1/2	6/2/2	
Pack-yr	0	19 ± 6	
Pulmonary function^4^			
FVC	106 ± 12	107 ± 13	
FEV1	103 ± 9	99 ±13	
FEV1/FVC	79 ± 4	76 ± 6	
TLC	90 ± 7	93 ±12	
DLCO	87 ± 7	89 ±5	
Epithelial cells^5^			
Number recovered x10^6^	1.2 ± 0.4	0.9 ± 0.3	0.9 ± 0.3
% epithelial cells	98.4 ± 0. 8	98.4 ± 0.7	98.4 ± 1.0
% inflammatory cells	1.6 ± 0.8	1.6 ± 0.7	1.7 ± 1.0
Differential cell count^6^			
Ciliated (%)	68.6 ± 6.2	66.9 ± 8.3	67.9 ± 6.5
Secretory (%)	9.1 ± 2.9	13.7 ± 6.8	13.7 ± 5.4
Basal (%)	13.6 ± 6.5	5.4 ± 4.9	3.9 ± 1.9
Undifferentiated (%)	7.1 ± 2.9	12.5 ± 6.9	12.8 ± 5.5

^1^ Data are presented as mean ± standard deviation

^2^ Criteria for quitter (inactive smoking stats) is defined as nicotine levels of <30 ng/ml and cotinine levels of <50 ng/ml for the urine tests.

^3^ B = Black, W = White, O = Other.

^4^ Pulmonary function testing parameters are given as % of predicted value with the exception of FEV1/FVC, which is reported as % observed; FVC—forced vital capacity; FEV1—forced expiratory volume in 1 sec; TLC—total lung capacity; DLCO—diffusing capacity.

^5^ Small airway epithelium was collected using fiberoptic bronchoscopy. To quantify the percentage of epithelial and inflammatory cells and the proportions of ciliated, basal, secretory, and intermediate epithelial cells, aliquots of 2x10^4^ cells were prepared by centrifugation and stained with DiffQuik.

^6^ Only epithelial cells were used for differential cell counting.

**Fig 1:**
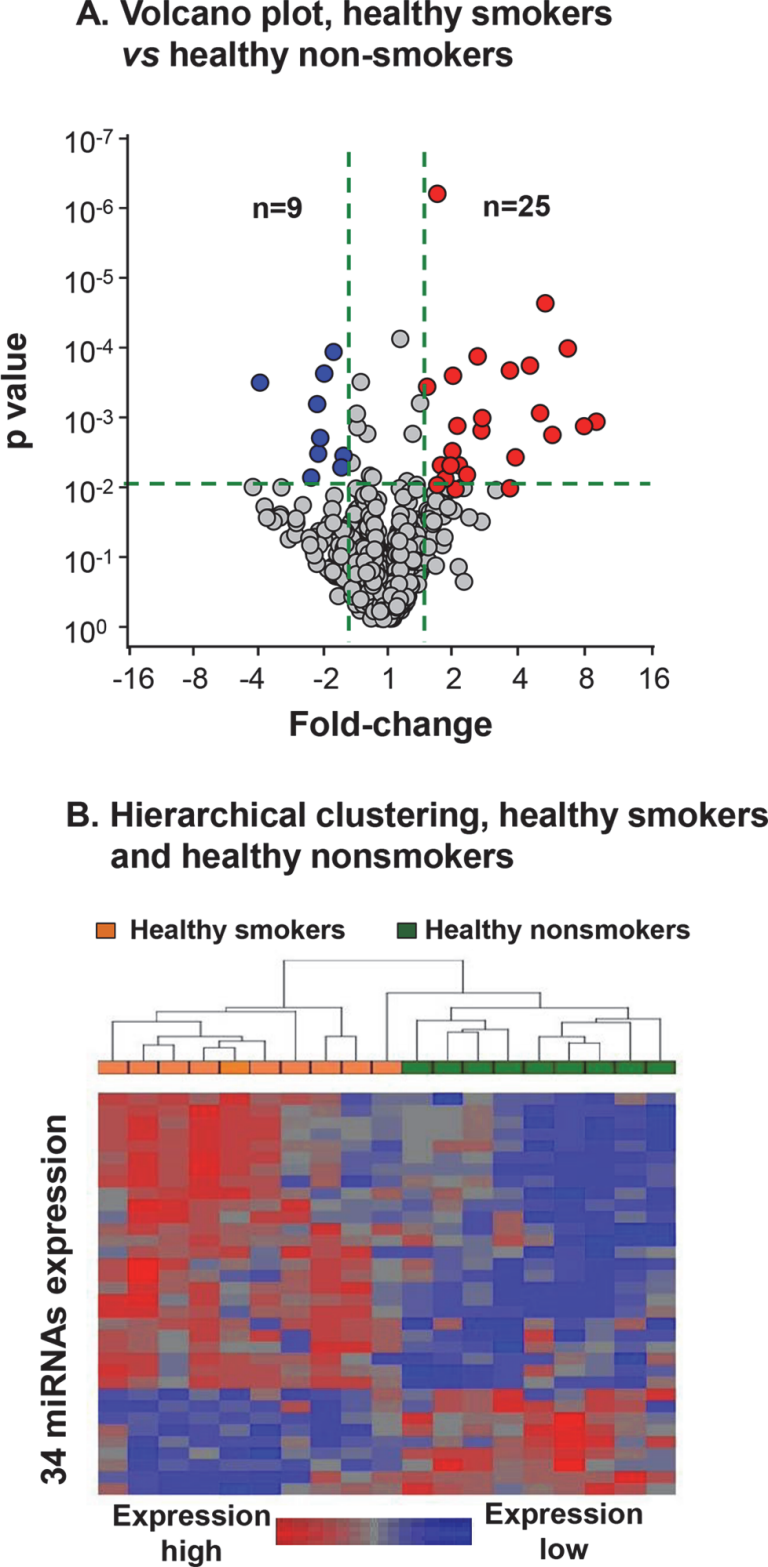
Volcano plot and unsupervised hierarchical clustering of differentially expressed miRNAs in the small airway epithelium (SAE) of healthy smokers and healthy nonsmokers. A. Volcano plot comparing the expression of 1100 human mature miRNAs in the SAE of smokers *vs* nonsmokers. There were 25 miRNAs (red) upregulated and 9 miRNAs (blue) downregulated in the SAE of healthy smokers compared to healthy nonsmokers (fold-change >1.5, p<0.01, total 34 miRNAs). X-axis, fold-change; Y-axis, p values. B. Unsupervised hierarchical clustering of 34 differentially expressed miRNAs between healthy smokers (tan rectangle) and healthy nonsmokers (green rectangle). The color bar indicates the relative miRNA expression level.

Using the criteria of p<0.01, fold-change>1.5, 34 out of 1100 human mature miRNAs were identified as significantly changed in the SAE by smoking (Fig 1A and Table 2). Unsupervised hierarchical clustering of the healthy smokers and healthy nonsmokers based on the 34 significantly expressed miRNAs separated healthy smokers and healthy nonsmokers into two distinct groups (Fig 1B). The majority of the smoking-dependent miRNAs were upregulated in healthy smokers compared to healthy nonsmokers (25 of 34 miRNAs, 74%; Fig 1A and Table 2). The miRNA that was most up-regulated in healthy smokers was miR-143 (p<10^–2^, 9.1-fold). The miRNA that was most down-regulated in healthy smokers was miR-1246 (p<10^–3^, -3.8-fold). Two miRNA families were over-expressed in healthy smokers compared to healthy nonsmokers, including the miR-181 family (miR-181a, miR-181b and miRNA-181c) [23] and the miR-133 family (miR-133a, miR-133b) [24]. Three microRNA clusters, miR-199a/miR-214 [25], miR-143/miR-145 [26] and miR-181a/miR-181b, were upregulated by smoking.

**Table 2:** miRNAs Significantly Up- and Down-regulated in the Small Airway Epithelium by Cigarette Smoking^1^.

	Healthy smokers *vs* healthy nonsmokers	
Probeset ID	Fold-change^2^	p value^3^
**Up-regulated**		
hsa-miR-143_st	9.1	1.0x10^-3^
hsa-miR-145_st	8.0	1.2x10^-3^
hsa-miR-133b_st	6.7	8.9x10^-5^
hsa-miR-214_st	5.7	1.6x10^-3^
hsa-miR-634_st	5.3	2.0x10^-5^
hsa-miR-126_st	5.0	7.6x10^-4^
hsa-miR-139-5p_st	4.5	1.6x10^-4^
hsa-miR-199a-3p_st	3.9	3.3x10^-3^
hsa-miR-199a-5p_st	3.7	9.1x10^-3^
hsa-miR-133a_st	3.7	1.9x10^-4^
hsa-miR-195_st	2.7	8.9x10^-4^
hsa-miR-181c_st	2.7	1.3x10^-3^
hsa-miR-675_st	2.6	1.2x10^-4^
hsa-miR-127-3p_st	2.3	5.8x10^-3^
hsa-let-7b-star_st	2.1	4.2x10^-3^
hsa-miR-1260_st	2.1	1.2x10^-3^
hsa-miR-1226-star_st	2.1	9.3x10^-3^
hsa-miR-636_st	2.0	2.2x10^-4^
hsa-miR-193b-star_st	2.0	2.6x10^-3^
hsa-miR-487b_st	2.0	4.3x10^-3^
hsa-miR-193b_st	1.9	6.6x10^-3^
hsa-miR-138-1-star_st	1.8	4.2x10^-3^
hsa-miR-181a_st	1.7	5.4x10^-7^
hsa-miR-550_st	1.7	8.2x10^-3^
hsa-miR-181b_st	1.5	3.1x10^-4^
**Down-regulated**		
hsa-miR-449b_st	-1.6	3.1x10^-3^
hsa-miR-224-star_st	-1.6	4.5x10^-3^
hsa-miR-1975_st	-1.8	1.4x10^-4^
hsa-miR-1979_st	-1.9	2.1x10^-4^
hsa-miR-218_st	-2.0	1.7x10^-3^
hsa-miR-146a_st	-2.1	2.9x10^-3^
hsa-miR-203_st	-2.1	5.6x10^-4^
hsa-miR-3201_st	-2.2	6.3x10^-3^
hsa-miR-1246_st	-3.8	2.7x10^-4^

^1^ Affymetrix miRNA 2.0 arrays.

^2^ Fold-change: healthy smokers *vs* healthy nonsmokers.

^3^ p value: healthy smokers *vs* healthy nonsmokers.

MicroRNA function enrichment analysis showed that the top 4 functions (p<0.05, ranked by p value) of the 25 up-regulated miRNAs were miRNA tumor suppressors (p<10^–4^, fold-enrichment 6.1), inflammation (p<10^–2^, fold-enrichment 3.4), human embryonic stem cell regulation (p<0.05, fold-enrichment 2.3) and cell differentiation (p<0.05, fold-enrichment 5, Table 3 and S2 Table). The top 4 functions of the 9 down-regulated miRNAs included inflammation (p<10^–2^, fold-enrichment 6.6), immune response (p<10^–2^, fold-enrichment 5.7), onco-miRNAs (p<0.05, fold-enrichment 5.8) and human embryonic stem cell regulation (p<0.05, fold-enrichment 3.2; Table 3 and S2 Table). The enriched functions of the smoking-dependent miRNAs are consistent with the knowledge that chronic smoking is associated with COPD and lung cancer [27].

**Table 3:** Top function categories of miRNAs with significantly different expression in the small airway epithelium of healthy smokers *vs* healthy nonsmokers^1^.

Category^2^	Count^3^	Percent^4^	Fold-enrichment	p value^5^
**25 up-regulated miRNAs**				
miRNA tumor suppressor	8	22	6.1	8.0x10^-6^
Inflammation	5	12	3.4	1.0x10^-2^
Human embryonic stem cell regulation	7	8	2.3	1.8x10^-2^
Cell differentiation	3	18	5.0	1.9x10^-2^
HIV latency	3	14	4.0	3.3x10^-2^
**9 down-regulated miRNAs**				
Inflammation	3	7	6.6	6.2x10^-3^
Immune response	3	6	5.7	9.3x10^-3^
Onco-miRNAs	2	6	5.8	4.1x10^-2^
Human embryonic stem cell regulation	3	4	3.2	4.9x10^-2^
Apoptosis	2	5	4.0	7.8x10^-2^

^1^ miRNA functional category was analyzed by “Tool for annotations of meaningful human miRNAs categories” (TAM; http://202.38.126.151/hmdd/tools/tam.html). The list of microRNAs for each category can be found in S1 Table.

^2^ TAM includes 24 miRNA function categories.

^3^ Count = number of miRNAs matched to the functional category.

^4^ Percent of matched miRNA/total number of miRNA in the function category.

^5^ Significance of enrichment.

As inflammation was a major function for both smoking up- and down-regulated microRNAs, we investigated whether several of the smoking up-regulated and down-regulated microRNAs might be directed toward a common inflammation-related pathway. By manually assessing the literature, 6 smoking dependent microRNAs were identified as being associated with NF-kappa B pathway [28,29] (S1 Fig). For example, smoking up-regulated miR-181b and miR-126, two miRNAs that can promote NF-kappa B indirectly; smoking up-regulated miR-143, a NF-kappa B target; smoking down-regulated miR-146a and miR-218, two NF-kappa B negative regulators; and smoking upregulated miR-199a, an inhibitor of the NF-kappa B pathway. Together, these data point out the complexity of miRNA smoking-related modulation of the SAE gene expression, with different miRNAs competing to activate or suppress the same pathway.

As it is known that smoking is associated with disordering of airway epithelium differentiation, we carried out literature data mining for the specific role of each smoking dependent microRNA in lung development and airway epithelium differentiation. Interestingly, smoking up-regulated two lung development-related miRNAs, miR-214 and miR-127 (p<10^–3^, 5.8-fold) [17,19]. Relevant to the pathogenesis of COPD, activating transcription factor 4 (ATF4)-mediated endoplasmic reticulum stress (ER stress) has been suggested to be a key regulator for COPD-relevant airway epithelial gene expression [30], and ATF4 is a validated target of miR-214 [31]. Smoking also down-regulated miR-449b (p<0.01, -1.6-fold), a miRNA associated with airway epithelial differentiation [32].

### Effect of Smoking Cessation on miRNA Expression

**Fig 2:**
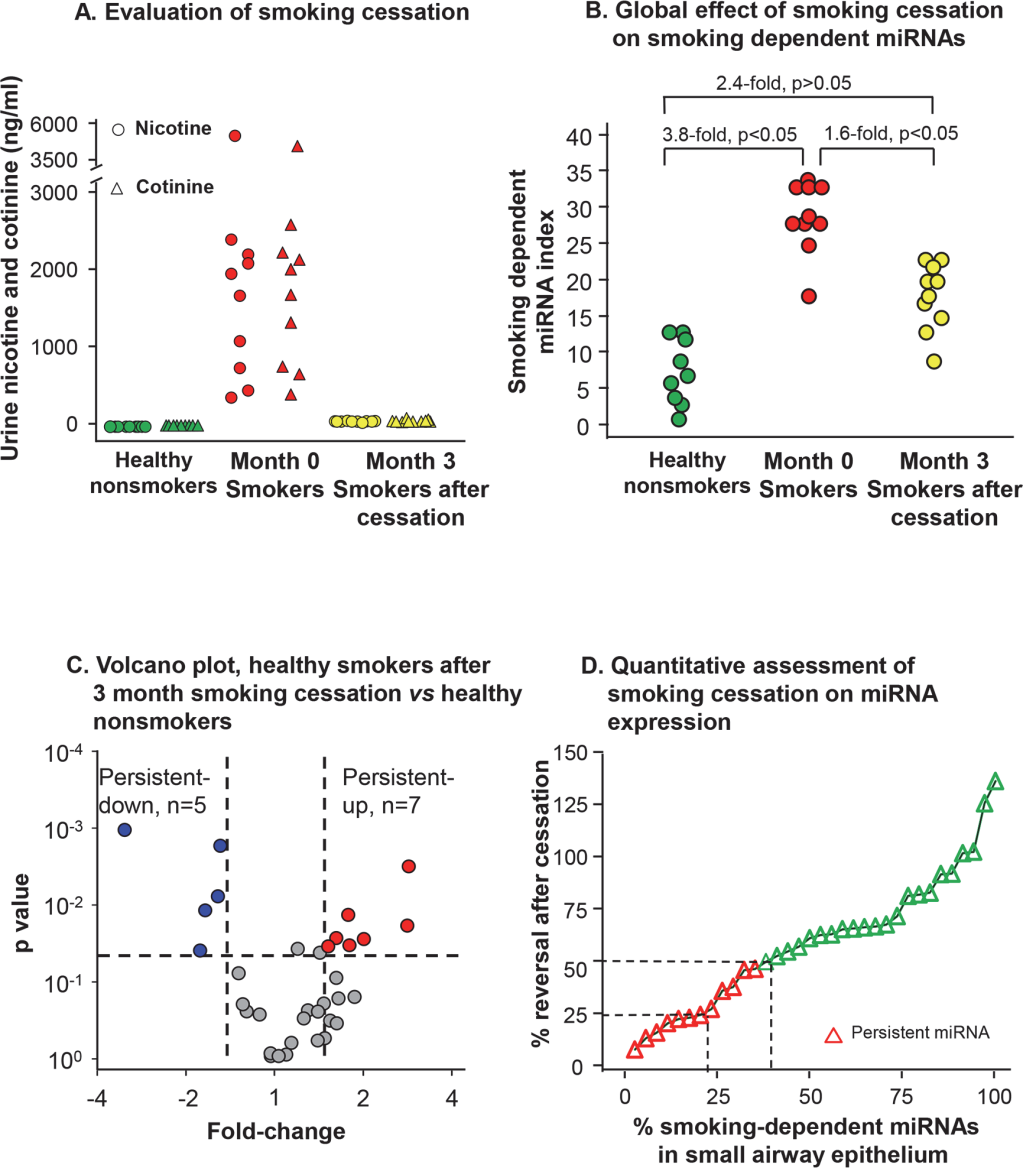
Effect of smoking cessation on the SAE expression of the 34 miRNAs dysregulated by smoking. A: Quantitative evaluation of smoking cessation. Urine nicotine and cotinine levels of healthy nonsmokers, healthy smoker before smoking cessation (month 0), healthy smokers after 3 months smoking cessation. B: Global effect of smoking cessation on smoking-dependent miRNAs. Smoking dependent miRNA indexes based on 34 smoking dependent miRNAs were calculated (see Methods) for each of the healthy nonsmokers, healthy smokers before smoking cessation (month 0) and healthy smokers after 3 months smoking cessation. C: Volcano plot of the 34 smoking-dependent miRNAs in healthy smokers after 3-month smoking cessation compared to healthy nonsmokers. X-axis, fold-change, healthy smokers after 3-month smoking cessation *vs* healthy nonsmokers; Y-axis, p value. Twelve of the 34 miRNAs were not reversed after smoking cessation (criteria p<0.05, fold-change >1.5). D: Quantitative assessment of smoking cessation on miRNA expression in human SAE. Using mean absolute expression levels, the degree of reversibility in a miRNA was calculated as: % change = [(healthy smokers before smoking cessation—healthy smokers after 3-month smoking cessation)x100 / (healthy smokers before smoking cessation—healthy nonsmokers)]. X-axis—percentage of the smoking dependent miRNA (in order of ascending % change). The persistent miRNA identified in panel C are shown as red. Y-axis—% change of miRNA. The vertical dash line indicates the percentage of miRNAs which have 25% and 50% reversal.

Assessment of urine nicotine and cotinine confirmed that the 10 smokers had quit, with urine nicotine and cotinine measured in the nonsmoking range at the 3 month assessment (Fig 2A). To assess the effect of smoking cessation on the expression of smoking-dependent miRNAs at a global level, a smoking-dependent miRNA index was used. Smoking up-regulated the smoking-dependent miRNA index (3.8-fold, p<0.05) and smoking cessation was associated with significant down-regulation of the index (1.6-fold, p<0.05, compared to healthy smokers, Fig 2B). When assessed at this global level of all smoking changed miRNAs, the differences between healthy nonsmokers and smokers that quit was not statistically significant, although the average miRNA index of the smoking cessation group was still 2.4-fold higher than that of the healthy nonsmokers, suggesting the levels of individual miRNAs may still be significantly abnormal even with smoking cessation.

Consistent with this concept, at the individual miRNA level, 22 out of the 34 smoking responsive miRNAs returned to the expression level of healthy nonsmokers (Fig 2C and Table 4). The other 12 miRNAs that did not return to the normal level represented 35% of the total number of differentially expressed miRNAs between healthy smokers and healthy nonsmokers (Fig 2C and Table 4). Quantitative assessment of the degree to which the expression of these miRNAs normalized to the level observed in healthy nonsmokers demonstrated that after 3 months smoking cessation, the expression of 21% of miRNAs reversed less than 25% and 38% of miRNAs reversed less than 50% (Fig 2D).

**Table 4:** miRNAs that remain significantly up- and down-regulated in the small airway epithelium despite cessation of cigarette smoking^1^.

	Healthy smokers after 3-month smoking cessation *vs* healthy nonsmokers	
Probeset ID	Fold-change^2^	p value^3^
**Persistently up-regulated**		
hsa-miR-634_st	2.8	3.0x10^-3^
hsa-miR-133b_st	2.8	1.8x10^-2^
hsa-miR-133a_st	2.0	2.7x10^-2^
hsa-miR-1226-star_st	1.8	3.2x10^-2^
hsa-miR-487b_st	1.8	1.3x10^-2^
hsa-miR-1260_st	1.6	2.6x10^-2^
hsa-miR-550_st	1.5	3.3x10^-2^
**Persistently down-regulated**		
hsa-miR-1246_st	-3.2	1.0x10^-3^
hsa-miR-3201_st	-1.8	3.8x10^-2^
hsa-miR-218_st	-1.7	1.1x10^-2^
hsa-miR-224-star_st	-1.6	7.4x10^-3^
hsa-miR-1975_st	-1.5	1.6x10^-3^

^1^ Affymetrix miRNA 2.0 arrays.

^2^ Fold-change: healthy smokers after 3-month smoking cessation *vs* healthy nonsmokers.

^3^ p value: healthy smokers after 3-month smoking cessation *vs* healthy nonsmokers.

Examples of “smoking cessation reversible miRNAs” included cancer and inflammation related miR-181a, airway epithelium differentiation related miR-449b, and lung development-related miR-214 and miR-127. Interestingly, many SAE “smoking cessation persistent miRNAs” have been associated with carcinogenesis and chronic airway disease. For example, the persistently-downregulated miRNA, miR-218, has been identified as a tumor suppressive miRNA in non-small cell lung cancer [33]. The persistently up-regulated miRNAs miR-133a and 133b has been reported to function as either onco-miRNA or tumor suppressive miRNA depending on cancer type [24,34]. The persistently up-regulated miR-487b has been associated with pulmonary fibrosis [35] and the persistently-downregulated miRNA, miRNA-1246, is a transcriptional target of P53 family genes [36]. Finally, mir-1246 itself can regulate expression of cystic fibrosis transmembrane conductance regulator (CFTR) [37].

### TaqMan PCR Validation of Differentially Expressed miRNAs

**Fig 3:**
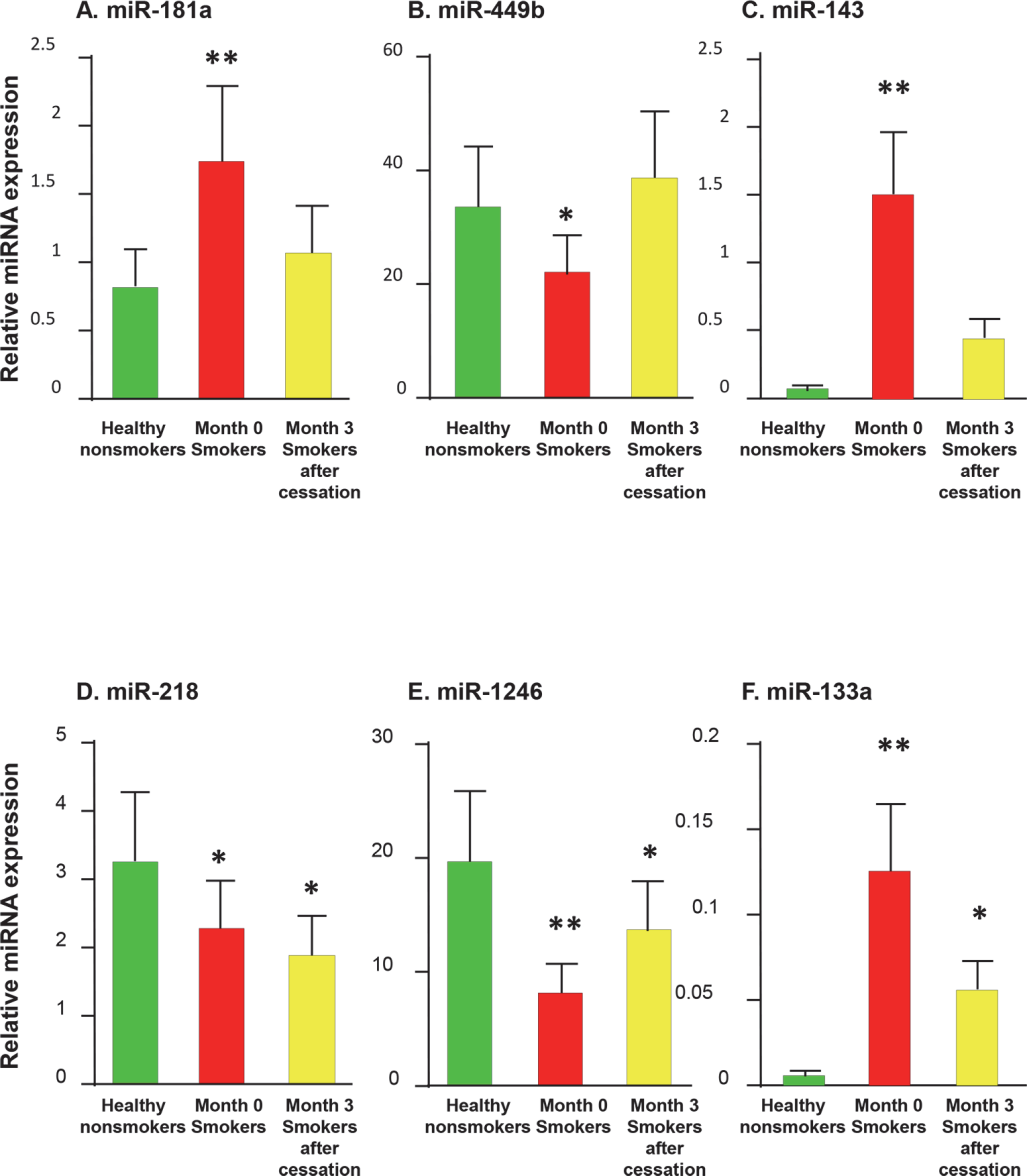
TaqMan quantitative PCR validation of smoking cessation reversible and smoking cessation persistent miRNAs in the SAE. MicroRNA let-7a was used as endogenous control. Error bars indicate standard error, and p values (compared with nonsmokers) were determined using Mann-Whitney test. *, p<0.05; **, p<0.01. **A-C:** Validation of reversible miRNAs after smoking cessation. **A:** miR-181a; **B:** miR-449b; and **C:** miR-143. **D-F:** Validation of persistent miRNAs after smoking cessation. **D:** miR-218; **E:** miR-1246; and **F:** miR-133a.

We performed confirmatory qRT-PCR for 13 microRNAs (3 reversible miRNAs, 10 persistent miRNAs) on all samples. The reversible smoking-induced expression changes of miR-181a, miR-449b and miR-143 (Fig 3A–3C), and the persistent changes of miR-218, miR-1246, miR-133a (Fig 3A–3C) and miR-634, miR-133b, miR-1226-star, miR-487b, miR-1260, miR-550 (S2 Fig) were verified by qRT-PCR. Two persistent miRNAs (miR-1975 and miR-3201) were not validated because no matched TaqMan Probes available. Probably because of limited sample size, we were not able to get significant changes of miR-1226 star by qRT-PCR (S2 Fig).

### Target Genes of Smoking Cessation Persistent miRNAs

**Fig 4:**
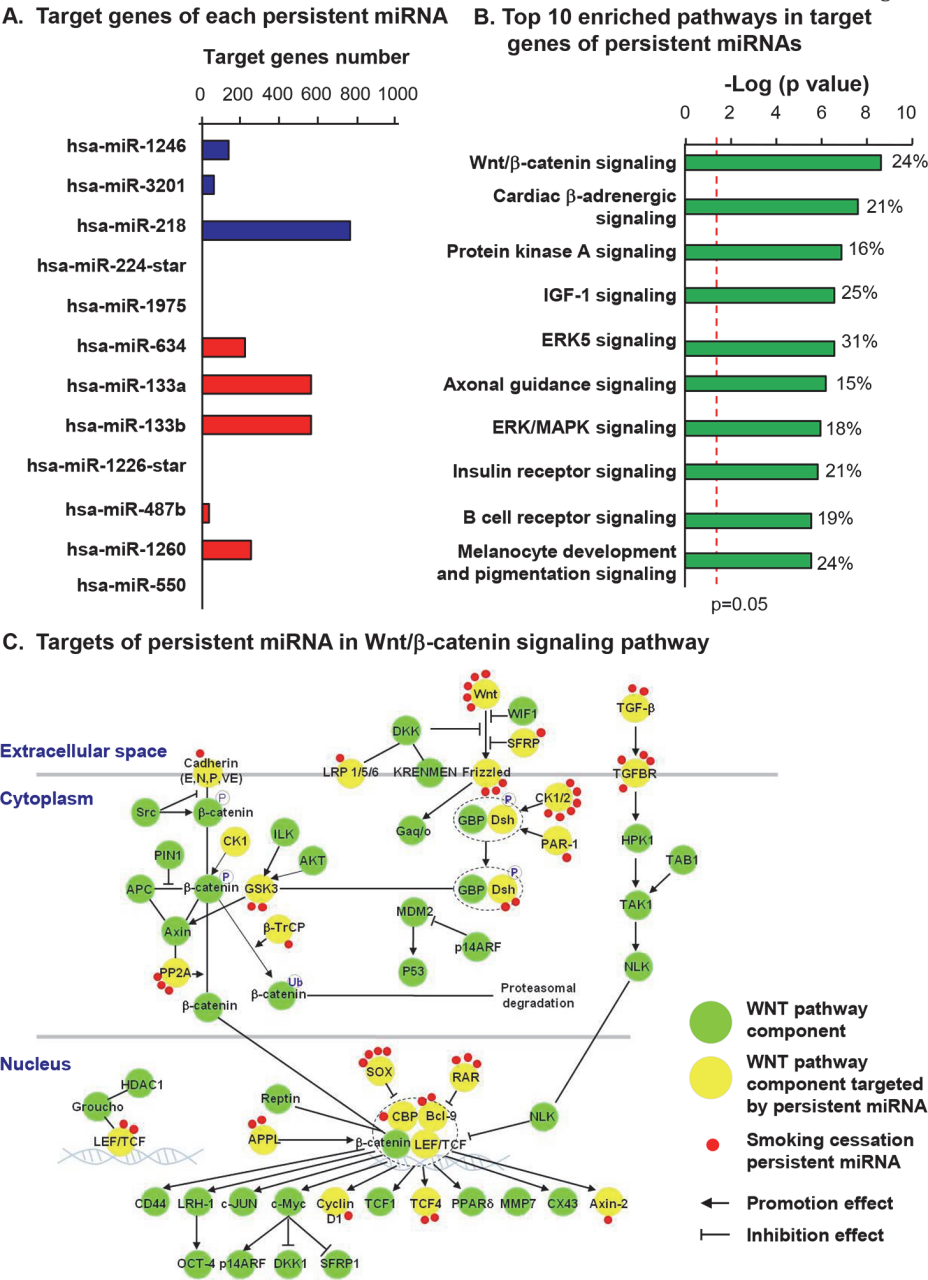
Target genes of smoking cessation persistently altered miRNAs in the human SAE. A: Numbers of predicted target genes for each of the persistent miRNAs. The target genes prediction was based on Target Scan 6.2 database. Only genes that are expressed in human SAE were used for the analysis. Blue, target genes of persistent-down-regulated miRNAs. Red, target genes of persistent-up-regulated miRNA. The persistently dysregulated miRNAs with the highest target gene number were miR-218 and miR-133a and miR-133b targets. B: Top 10 enriched canonical pathways in the target genes of the smoking cessation persistent miRNAs. The analysis was performed using Ingenuity Pathway Analysis software. X axis,-log of p value, Fisher's exact test. The ratio of genes that were targeted by the smoking cessation persistent-miRNA in each pathway are indicated. C: Wnt/β-catenin signaling pathway associated with persistently dysregulated miRNAs despite smoking cessation. The Wnt pathway genes that are targets of the smoking cessation persistent miRNAs are highlighted by yellow. The correspondent miRNAs are marked red, with the number of red dots corresponding to the number of miRNAs targeted toward each gene. Many Wnt pathway ligands, receptors, effectors and regulators are potential targets of the smoking cessation persistent miRNAs. Abbreviations: IGF-1, insulin-like growth factor 1; ERK5, Extracellular signal-regulated kinase 5; MAPK, mitogen-activated protein kinase.

To further assess the roles of smoking cessation persistent miRNAs in the SAE, the functions of their target genes were explored. Only those target genes known to be expressed in SAE were used for analysis [21]. There were 2,112 genes (excluding duplicates) that were predicted-targets of the smoking cessation persistent-miRNAs. Among these genes, 1763 were expressed in the human SAE. Interestingly, many of the target genes were targets of miR-218 and miR-133a/b (Fig 4A). By pathway analysis, Wnt/β-catenin signaling, cardiac β-adrenergic signaling and protein kinase A signaling were found to be the top 3 canonical pathways (ranked by p value) enriched in the target genes of smoking cessation persistent-miRNAs (Fig 4B and S3 Table). The Wnt/ β-catenin signaling pathway was the top enriched pathway, consistent with smoking being associated with dysregulated differentiation, lung cancer and inflammation. Remarkably, 24% of Wnt pathway genes were predicted to be targets of smoking cessation miRNAs, with some Wnt pathway genes targeted by multiple miRNAs, including Wnt ligands, receptors, regulators and effectors (Fig 4C).

To ask whether the smoking-deregulated miRNAs could have biologic effect *in vivo*, we analyzed our recently published SAE mRNA microarray dataset [22] and focused on the effect of smoking on Wnt pathway target genes of the persistent miRNAs. Interestingly, expression of target genes of the persistent miRNAs clearly segregated smokers and nonsmokers into two groups (PCA analysis, S3 Fig). The data suggests that smoking induces dysregulation of Wnt pathway in SAE and that persistent miRNAs might be involved during this process.

### Assessment of Target Genes of Persistent miRNAs

To assess putative target genes of the dysregulated-miRNA, microRNA inhibitors were used. We focused on miR-1246, which is a smoking-persistent-down microRNA validated by TaqMan PCR. Suppression of miR-1246 by microRNA inhibitor in primary human airway epithelial cells significantly up-regulated several putative miR-1246-target genes, including DRK1A, GRHL1 and GSK3B (S4 Fig).

## Discussion

The focus of this study was on the effect of smoking on miRNA levels in the SAE, the site of development of the smoking-related diseases, COPD and most types of lung cancer [2,3]. We identified 34 smoking-responsive miRNAs in the SAE, with functions associated with lung development, airway epithelium differentiation, inflammation and cancer. Interestingly, among these 34 miRNAs, 12 did not return to normal levels after 3 months of cigarette smoking cessation. The functions of many of these smoking cessation persistent miRNAs and /or their target mRNAs have been associated with pathogenic processes linked to chronic airway disease and/or lung cancer [24,33–35,37,38]. This is consistent with the knowledge that while smoking cessation improves respiratory symptoms and bronchial hyper-responsiveness, delays the decline in FEV1 compared to continuing smokers, and reduces the risk for lung cancer [7,39,40], the risk for COPD and lung cancer remains significantly higher compared to healthy nonsmokers despite smoking cessation [7,8].

### miRNAs in Lung Development and Airway Epithelium Differentiation

miRNA profiling in lung development has identified a number of miRNAs differentially expressed during different stages of lung development, with similarities in studies of the developing mouse, rat and human lung [17–19]. Consistent with these observations, and the knowledge that smoking is associated with disordering of airway epithelial differentiation [3,4], several of the miRNAs differentially expressed in the smoker SAE have been implicated as important in lung development. For example, smoking up-regulates the expression of miR-214, miR-127, miR-145 and down-regulates miR-449b in the SAE. miR-214 levels change significantly in mouse and rat lung development [17,19], while miR-127 overexpression is associated with a decrease in lung terminal bud count and increase in internal and terminal bud sizes [20]. miR-449b, a miRNA down-regulated in the smoker SAE, has been shown to enhance cilia biosynthesis in human airway epithelium by repressing the delta/notch pathway [32], and is down-regulated in the airway epithelium in asthma [41]. miR-145, a miRNA up-regulated in the SAE by smoking, plays a role in human embryonic stem cells [42], and targets SOX2 and KLF4, both important transcription factors in human airway epithelium [21]. The dysregulation of miR-214, miR-127, miR-449b and miR-145 in the SAE is reversed by smoking cessation, consistent with the knowledge that smoking cessation does reverse many of the smoking-related epithelial lung pathologies [43].

### Expression of miRNAs by Different Lung Cell Populations

The SAE, the cell population central to the initial pathology of the smoking-related lung disorders, represents, at most, 2% of the total population of the lung parenchyma [44], and thus likely does not contribute significantly to miRNA analyses of samples of lung parenchyma *per se*. With this caveat, there are interesting parallels between the observations in the present study and studies of miRNA in pieces of lung parenchyma in health and disease. For example, in the SAE, we observed that smoking down-regulated miR-146a. Of interest, miR-146a, a miRNA that targets COX2 and suppresses arachidonic acid metabolism, is up-regulated in lung fibroblasts by cytokines, but less so in fibroblasts derived from the COPD lung [45]. We also observed that smoking up-regulates miR-199a-5p in the SAE, a miRNA that targets hypoxia-inducible factor-1α (HIF-1α) and suppresses HIF-1α in human pulmonary microvascular endothelial cells. miR-199a-5p is up-regulated in COPD lung tissue [46]. Globally, the Wnt pathway is enriched as targets of the smoking cessation persistent miRNAs in SAE. Interestingly, the Wnt pathway has also been identified as a major target of dysregulated-miRNAs in a COPD lung tissue-based study [47]. As most COPD patients are smokers, these similarities suggest some common smoking-related miRNA responses may be present in different cell populations in the lung.

Finally, our data suggests that there are differences in miRNA regulation in the large airway compared to the SAE. In this regard, Schembri et al [20] examined miRNA expression from human large airway epithelial cells of healthy smokers and healthy nonsmokers and found 28 miRNAs to be significantly differentially expressed with the majority being down-regulated in smokers. The most significantly down-regulated miRNA in the large airway epithelium of smokers compared to healthy nonsmokers was miRNA-218 [20]. Although we also observed miR-218 to be down-regulated in the SAE, most of the smoking-dependent miRNAs we found in SAE were not paralleled in the large airway. Further, unlike the dominant down-regulation of miRNA expression in large airway epithelium [20], there were more miRNAs up-regulated in the SAE from healthy smokers. Further study focusing on microRNAs from different airway regions of same individuals might explain this observation.

### Small Airway Epithelium miRNA Levels Following Smoking Cessation

The reversibility of miRNAs targeting NF-kappa B [28,29], COX2 [45], HIF-1α [46], ER stress [31] and Notch pathway [32] is consistent with the role of smoking cessation in improving respiratory symptoms. However, even after 3 months smoking cessation, some of the dysregulation of SAE miRNAs persisted. Some, like miR-133a, miR-133b, miR-487b, miR-1246 and miR-218, are cancer or inflammation-related miRNAs [24,33–35,37]. Of note, miR-487b can be regulated by DNA methylation [48], and miR-218 is a tumor repressor in several types of cancers, including lung carcinoma [33]; miR-1246 is a downstream gene of the p53 oncogene [36]. Globally, the Wnt pathway is enriched in the target genes of the persistent miRNAs, consistent with the knowledge that Wnt pathway dysregulation is associated with chronic lung diseases, including lung cancer and COPD [49,50].

The reasons for the persistent changes of miRNAs with smoking cessation are not clear. Possible mechanisms include the persistence of cigarette smoke-related components in the lung tissue, persistent airway inflammation [51,52], changes in airway epithelial cell populations, persistent changes of transcription factors controlling miRNAs, or genomic or epigenetic changes directly affecting miRNA expression [53]. Another interesting question is whether disordered-airway basal stem/progenitor cells contribute to these persistent miRNAs. The healthy airway epithelial cell population is renewed every 3 months [54], and it takes approximately 30 days to get fully differentiated airway epithelium from human basal cells *in vitro*, a time frame within our 3 month smoking cessation study period.

### Therapeutic Prospect of Persistent miRNAs in Airway Disease

To some extent, smoking cessation is the best “drug” to prevent smoking-induced airway diseases like COPD and lung cancer. However, even after smoking cessation, some airway epithelial molecular changes persist, including dysregulation of some miRNAs. Encouraged by progress in the human trials of targeting miR-122 for the treatment of the hepatitis C virus [55], it is tempting to speculate that the persistent dysregulated-miRNAs would be candidates for drug development to prevent smoking-related lung disorders. The accessibility of the airway to topical administration makes this approach even more attractive. However, there are still several concerns need to be addressed. For example, it still remains unclear whether these microRNAs will persist for even longer time and whether the persistent change is simply a reflection of the disease or an essential mediator of the underlying pathology. Since the pathogenesis of airway disease like COPD is very complex with the involvement of multiple cells types, more mechanism investigations, including animal studies, are warranted to gain more insight into the role of these dysregulated miRNAs.

## Supporting Information

S1 Fig (TIF)

S2 Fig (TIF)

S3 Fig (TIF)

S4 Fig (TIF)

S1 Methods (PDF)

S1 Table TaqMan microRNA Assays Used to Validate the Microarray Data^1^. (PDF)

S2 Table Functional Categories of Smoking-dependent microRNAs^1,2^. (PDF)

S3 Table Top 10 Canonical Pathways Used by the Target Genes of the Smoking Cessation Persistent miRNAs. (PDF)
