# Supplementary figures and images for: Persistence of Smoking-Induced Dysregulation of MiRNA Expression in the Small Airway Epithelium Despite Smoking Cessation
Source: PLoS One. 2015 Apr 17;10(4):e0120824. doi: 10.1371/journal.pone.0120824 (PMC4401720; doi:10.1371/journal.pone.0120824)

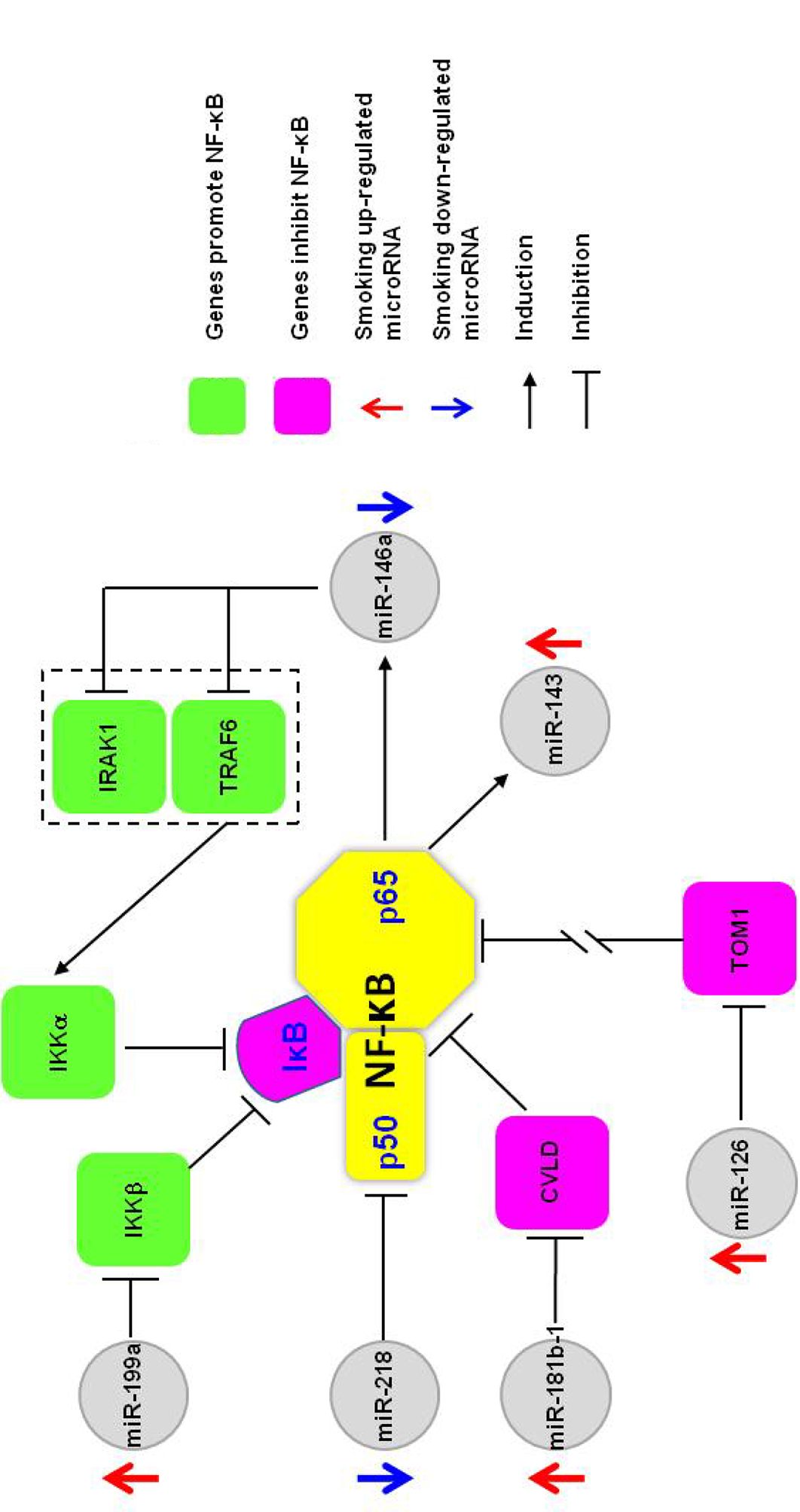

Supplement: S1 Fig — (TIF) [file pone.0120824.s001.tif]

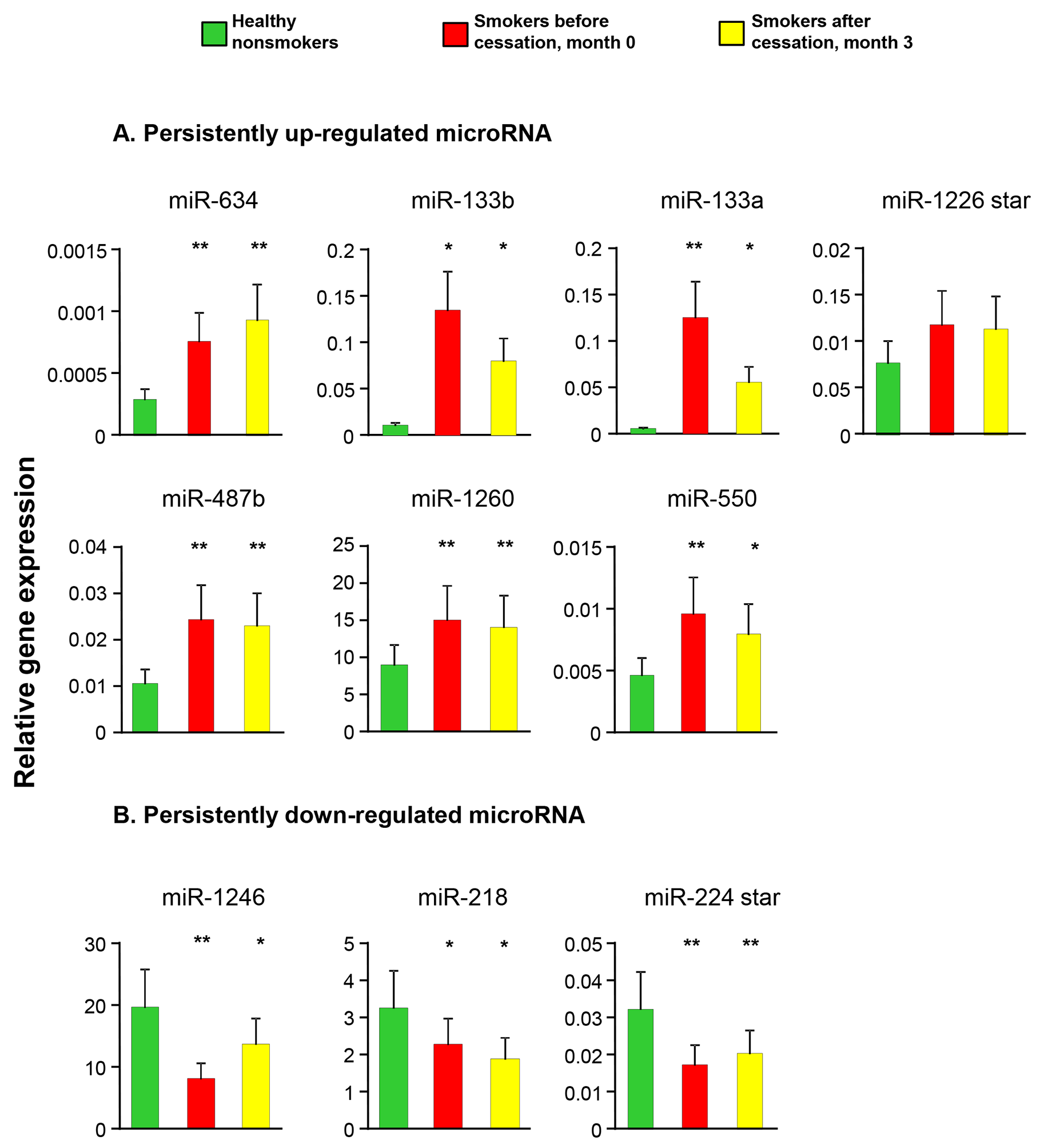

Supplement: S2 Fig — (TIF) [file pone.0120824.s002.tif]

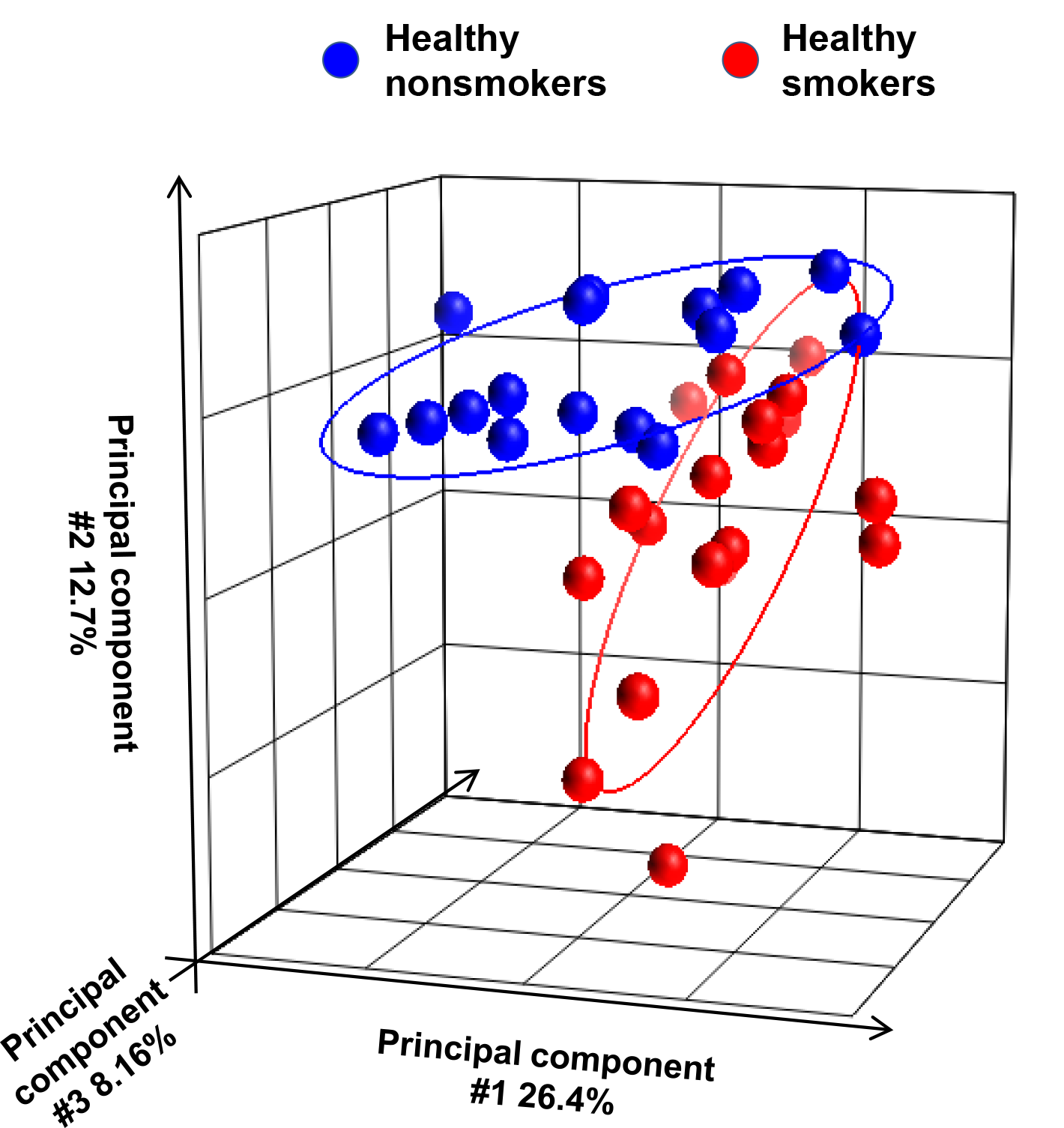

Supplement: S3 Fig — (TIF) [file pone.0120824.s003.tif]

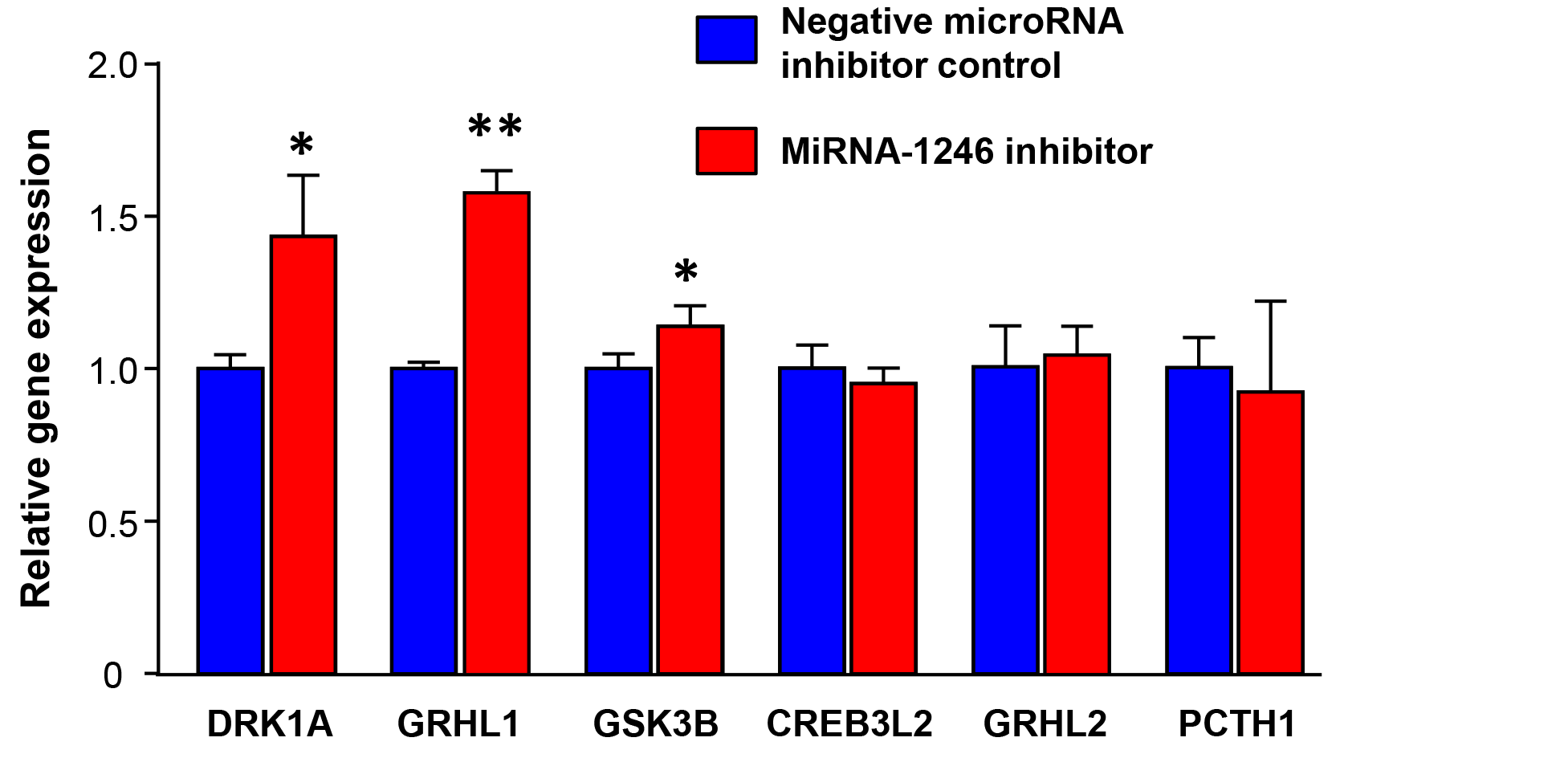

Supplement: S4 Fig — (TIF) [file pone.0120824.s004.tif]
