# Supplementary material for: Persistence of Smoking-Induced Dysregulation of MiRNA Expression in the Small Airway Epithelium Despite Smoking Cessation
Source: PLoS One. 2015 Apr 17;10(4):e0120824. doi: 10.1371/journal.pone.0120824 (PMC4401720; doi:10.1371/journal.pone.0120824)
Supplement: S1 Methods — (PDF) [file pone.0120824.s005.pdf]

Supporting Methods

miRNA Extraction and Microarray Processing

For the miRNA array, total RNA was extracted using miRNeasy mini kit (Qiagen, Valencia, CA) following the manufacture's protocol. RNA concentration was measured with Nanodrop ND-1000 spectrophotometer (Thermo Scientific, Wilmington, DE). RNA integrity was assessed on an Agilent Bioanalyzer (Agilent Technologies, Santa Clara, CA). The RNA Integrity Numbers (RINs) were >5.5 for all samples. Total RNA 0.5 μg was biotin labeled with the FlashTag® HSR RNA labeling kit (Genisphere, Hatfield, PA) and then hybridized to Affymetrix miRNA 2.0 arrays (Affymetrix, Santa Clara, CA) according to the manufacture's protocol. miRNA QC tool (Affymetrix) was used for quality control.

The image files from Affymetrix miRNA 2.0 microarrays were processed in Partek Genomics Suite software version 6.6, 2012 (Partek Inc, St. Louis, MO). A list of human mature miRNAs (miRBase V15 only, n=1100) were used for the analysis. Robust microarray analysis (RMA) algorithm was used for normalization. Two way ANOVA (gender was identified as a source of variation) followed by Fisher's Least Significant Difference test was used to identify the differentially expressed miRNAs among groups. Statistical significance was defined by $p<0.01$ and fold-change >1.5 for comparison of healthy smokers vs healthy never-smokers; and $p<0.05$ and fold-change >1.5 for comparison of healthy never-smokers *vs* healthy smokers after 3-month smoking cessation.

Inclusion and Exclusion Criteria

Healthy nonsmokers

Inclusion criteria

- Males and females, at least 18 years old
- Provide informed consent
- Normal physical examination
- Normal routine laboratory evaluation, including general hematologic studies, general serologic/immunologic studies, general biochemical analyses, and urine analysis
- HIV negative
- Not pregnant (females)
- Normal PA and lateral chest X-ray
- Normal electrocardiogram (sinus bradycardia, premature atrial contractions are permissible)
- Good health without history of chronic lung disease, including asthma, and without recurrent or recent (within 3 months) acute pulmonary disease
- Not taking any medications relevant to lung disease or having an effect on the airway epithelium
- No history of allergies to medications used in the bronchoscopy procedure
- Willingness to participate in the study
- Self-reported nonsmokers, with smoking status validated by the absence of nicotine and cotinine in urine (nicotine <30 ng/ml and cotinine <50 ng/ml)

Exclusion criteria

- Unable to meet the inclusion criteria
- Evidence of malignancy within the past 5 years
- Current active infection or acute illness of any kind
- Alcohol or drug abuse within the past 6 months

Healthy smokers

Inclusion criteria

- Males and females, at least 18 years old
- Provide informed consent
- Normal physical examination
- Normal routine laboratory evaluation, including general hematologic studies, general serologic/immunologic studies, general biochemical analyses, and urine analysis
- HIV negative
- Not pregnant (females)
- Normal PA and lateral chest X-ray
- Normal electrocardiogram (sinus bradycardia, premature atrial contractions are permissible)
- Good health without history of chronic lung disease, including asthma, and without recurrent or recent (within 3 months) acute pulmonary disease
- Not taking any medications relevant to lung disease or having an effect on the airway epithelium
- No history of allergies to medications used in the bronchoscopy procedure

- Willingness to participate in the study
- Self-reported current smokers with a minimum of 15 pack-yr smoking history, with current smoking status validated by urine cotinine level ≥104 ng/ml at baseline bronchoscopy [1]
- The subjects underwent smoking cessation for 3 months and had no urine metabolites in their urine at the month 3 bronchoscopy assessment (nicotine <30 ng/ml and cotinine <50 ng/m)

Exclusion criteria
- Unable to meet the inclusion criteria
- Evidence of malignancy within the past 5 years
- Current active infection or acute illness of any kind
- Alcohol or drug abuse within the past 6 months

Supporting References

1. Strulovici-Barel Y, Omberg L, O'Mahony M, Gordon C, Hollmann C et al. Threshold of biologic responses of the small airway epithelium to low levels of tobacco smoke. Am J Respir Crit Care Med. 2010;182: 1524-1532.

2. Boldin MP, Baltimore D. MicroRNAs, new effectors and regulators of NF-kappaB. Immunol Rev. 2012;246: 205-220.

3. Ma X, Becker Buscaglia LE, Barker JR, Li Y. MicroRNAs in NF-kappaB signaling. J Mol Cell Biol. 2011;3: 159-166.
