# Supplementary material for: Persistence of Smoking-Induced Dysregulation of MiRNA Expression in the Small Airway Epithelium Despite Smoking Cessation
Source: PLoS One. 2015 Apr 17;10(4):e0120824. doi: 10.1371/journal.pone.0120824 (PMC4401720; doi:10.1371/journal.pone.0120824)
Supplement: S1 Table — (PDF) [file pone.0120824.s006.pdf]

S1 Table. TaqMan microRNA Assays Used to Validate the Microarray Data[1]

microRNA	TaqMan MicroRNA assay catalog number
miR-181a	000480
miR-449b	001408
miR-143	002249
miR-634	001576
miR-133b	002247
miR-133a	002246
miR-1226 star	002758
miR-487b	001285
miR-1260	002896
miR-550	002410
miR-1246	462575_mat
miR-218	000521
miR-1246	462575_mat
miR-224 star	121210_mat
RNU6B	001093

[1] Life Technologies, Grand Island, NY
