# Supplementary material for: Persistence of Smoking-Induced Dysregulation of MiRNA Expression in the Small Airway Epithelium Despite Smoking Cessation
Source: PLoS One. 2015 Apr 17;10(4):e0120824. doi: 10.1371/journal.pone.0120824 (PMC4401720; doi:10.1371/journal.pone.0120824)
Supplement: S2 Table — (PDF) [file pone.0120824.s007.pdf]

S2 Table. Functional Categories of Smoking-dependent microRNAs[1,2]

Function category	microRNAs
**Up-regulated miRNAs**	
miRNA tumor suppressor	miR-181a, miR-181b, miR-181c, miR-127, miR-145, miR-143, miR-195, miR-126
Inflammation	miR-199a, miR-133a, miR-143, miR-126, miR-181a
Human embryonic stem cell regulation	miR-181a, miR-126, miR-199a , miR-214, miR-145, miR-143, miR-195
Cell differentiation	miR-145, miR-143, miR-127
HIV latency	miR-143, miR-126, miR-127
**Down-regulated miRNAs**	
Inflammation	miR-203, miR-146a, miR-449b
Immune response	miR-203, miR-146a, miR-449b
Onco-miRNAs	miR-203, miR-146a
Human embryonic stem cell regulation	miR-203, miR-146a, miR-218
Apoptosis	miR-146a, miR-449b

[1] miRNA functional category was analyzed (before December, 2012) by “Tool for annotations of meaningful human miRNAs categories” (TAM; http://202.38.126.151/hmdd/tools/tam.html).

[2] See Table III in the main text for more information.
