# Supplementary material for: Persistence of Smoking-Induced Dysregulation of MiRNA Expression in the Small Airway Epithelium Despite Smoking Cessation
Source: PLoS One. 2015 Apr 17;10(4):e0120824. doi: 10.1371/journal.pone.0120824 (PMC4401720; doi:10.1371/journal.pone.0120824)
Supplement: S3 Table — (PDF) [file pone.0120824.s008.pdf]

S3 Table. Top 10 Canonical Pathways Used by the Target Genes of the Smoking Cessation Persistent miRNAs

Ingenuity canonical pathways[1]	p value[2]	Ratio[3] (%)	Target genes of the smoking cessation persistent miRNAs in the given pathway[4]
Wnt/β-catenin signaling	10^{-9}	24	TCF4,SFRP2,TGFBR1,WNT10B,CSNK1G1,PPP2CA,PPP2R2A,TGFBR3,MARK2,LRP6,SOX12,BCL9,CCND1,TCF7,CSNK1E,RARA,RARB,TGFB2,WNT4,GSK3B,CSNK2B,SOX5,SOX4,AXIN2,APPL2,CSNK1G3,PPP2R5D,WNT2B,CREBBP,ACVR1B,PPP2R5A,FZD8,PPP2CB,CDH2,FZD4,WNT3A,SOX6,PPP2R4,BTRC,DVL3,PPP2R5E
Cardiac β -adrenergic signaling	10^{-8}	21	AKAP12,PPP1CC,PDE7A,PPP2R2A,PPP2CA,PDE3A,PDE12,PPP1CB,ATP2A2,AKAP11,GNB1,GNB4,ADRB1,ADCY5,ADCY9,CACNA1D,PDE10A,ADRBK2,PPP2R5D,ADCY6,AKAP6,PPP1R11,GNG3,PPP2R5A,PDE8A,PPP2CB,AKAP2/PALM2,AKAP2,PRKAR2B,PPP2R4,PRKACA,PDE8B,PPP2R5E AKAP9
Protein kinase A signaling	10^{-7}	16	TGFBR1,MAPK1,NFATC3,PDE12,PDE3A,CREB5,TCF7,GNB1,GNB4,PTPRO,ADCY5,DUSP7,GSK3B,ADCY9,YWHAG,YWHAE,PDE10A,PTCH1,CREBBP,YWHAZ,PPP1R11,PLCL2,GNG3,PTPN3,PTP4A1,PTPRM,PDE8A,PTPN11,PRKACA,PTPRA,CAMK2G,AKAP12,PPP1CC,TCF4,PDE7A,PPP1CB,AKAP11,DUSP5,NFAT5,CREB1,TGFB2,PRKCE,PRKCA,PXN,PTPRK,MAP3K1,ADCY6,PLCG1,AKAP6,NFATC1,GNAI2,GNAI3,CALM1,AKAP2/PALM2-AKAP2,PTPRU,PRKAR2B,FLNC,CDC14B,PDE8B,SFN,ELK1,EYA1,AKAP9,PTPRT,DUSP16
IGF-1 signaling	10^{-7}	25	SOCS3,CTGF,MAPK1,PIK3R1,SOCS6,PDPK1,PIK3CG,IGF1R,CSNK2B,RPS6KB1,PXN,NRAS,YWHAG,PIK3C2A,YWHAE,GRB2,YWHAZ,SOCS4,PRKAR2B,PTPN11,IRS1,PRKACA,ELK1,SOCS7,SFN,SOCS5
ERK5 signaling	10^{-7}	31	RPS6KB1,YWHAG,NRAS,YWHAE,SGK1,CREBBP,RPS6KA3,YWHAZ,CREB5,RPS6KA6,GAB1,PTPN11,MEF2D,CREB1,MEF2C,SFN,MAP3K3,ELK4,MAP3K2,EGFR
Axonal guidance signaling	10^{-6}	15	WNT10B,MAPK1,ITSN1,NFATC3,PIK3R1,UNC5B,ARPC5,SEMA6B,SEMA4F,BRCC3,GNB1,GNB4,SUFU,PIK3CG,WNT4,PLXNB2,SRGAP2,GSK3B,EPHA7,KALRN,CRKL,SEMA5A,PTCH1,GNG3,PLCL2,SRGAP3,PTPN11,PRKACA,GNAL,NRP1,ADAM17,RGS3,ARPC1B,SEMA6A,CRK,PLXNA2,ROBO1,EIF4E,EFNB2,NFAT5,MYSM1,PLXNA1,SDC2,EFNB1,MKNK1,PRKCE,PFN2,RASSF5,SHANK2,PRKCA,TUBB1,EPHB4,PXN,NRAS,PLXNC1,ARHGEF12,NRP2,PIK3C2A,GRB2,WNT2B,PLCG1,NFATC1,GNAI2,GNAI3,SEMA3A,FZD8,PRKAR2B,FZD4,WNT3A

S3 Table. Top 10 Canonical Pathways Enriched in the Target Genes of the Smoking Cessation Persistent miRNAs (cont., page 2)

Ingenuity canonical pathways[1]	p value[2]	Ratio[3] (%)	Target genes of the smoking cessation persistent miRNAs in the given pathway[4]
ERK/MAPK signaling	10^{-6}	18	PPP1CC,MAPK1,PPP2R2A,PPP2CA,PIK3R1,ETS2,PPP1CB,CRK,CREB5,EIF4E,ELF3,PIK3CG,MKNK1,CREB1,PRKCE,PRKCA,ETS1,PXN,YWHAG,NRAS,PIK3C2A,GRB2,PPP2R5D,CRKL,CREBBP,YWHAZ,PLCG1,NFATC1,PPP1R11,PPP2R5A,PPP2CB,ELF2,PRKAR2B,ARAF,PPP2R4,PRKACA,PPP2R5E,ELK1
Insulin receptor signaling	10^{-6}	21	SOCS3,PPP1CC,MAPK1,SGK1,PIK3R1,PDPK1,PPP1CB,CRK,STXBP4,EIF4E,PIK3CG,GSK3B,SCNN1B,RPS6KB1,NRAS,PIK3C2A,GRB2,CRKL,PPP1R11,VAMP2,PRKAR2B,RHOQ,CBL,PTPN11,SYNJ1,GAB1,IRS1,ASIC1,PRKACA,INSR
B cell receptor signaling	10^{-6}	19	MAP2K4,GAB2,MAPK1,NFATC3,POU2F2,PIK3R1,PDPK1,CREB5,NFAT5,PIK3CG,CREB1,GSK3B,MAP3K2,ETS1,RPS6KB1,NRAS,PIK3C2A,GRB2,MAP3K13,CREBBP,MAP3K1,NFATC1,BCL2L1,CALM1 (includes others),PTPN11,SYNJ1,GAB1,VAV3,PAG1,PIK3AP1,ELK1,MAP3K3,CAMK2G

[1] The pathway analysis was performed in Ingenuity pathway analysis software. The target genes of the 12 smoking cessation persistent miRNAs were used. See methods for detail. The pathways were ranked by p value.

[2] Fisher's exact test.

[3] Number of target genes in a given pathway divided by total number of genes that make up that pathway.

[4] Only target genes of the smoking cessation persistent miRNAs in a given pathway were shown.
